# Regulatory delays in a multinational clinical stroke trial

**DOI:** 10.1177/23969873211004845

**Published:** 2021-03-30

**Authors:** Jeroen C de Jonge, Hendrik Reinink, Bridget Colam, Iris Alpers, Alfonso Ciccone, Laszlo Csiba, Janika Kõrv, Iwona Kurkowska-Jastrzebska, Malcolm R Macleod, George Ntaios, Götz Thomalla, Philip M Bath, H Bart van der Worp

**Affiliations:** 1 Department of Neurology and Neurosurgery, University Medical Center Utrecht Brain Center, Utrecht University, Utrecht, The Netherlands; 2 Centre for Clinical Brain Sciences, University of Edinburgh, Edinburgh, UK; 3 Clinical Trial Center North GmbH, University Medical Center Hamburg-Eppendorf, Hamburg, Germany; 4 Department of Neurology and Stroke Unit, ASST di Mantova, Mantua, Italy; 5 Department of Neurology, University of Debrecen, Debrecen, Hungary; 6 Department of Neurology and Neurosurgery, University of Tartu, Tartu, Estonia; 7 2nd Department of Neurology, Institute of Psychiatry and Neurology, Warsaw, Poland; 8 Department of Internal Medicine, Faculty of Medicine, School of Health Sciences, University of Thessaly, Larissa, Greece; 9 Department of Neurology, Center for Clinical Neurosciences, University Medical Center Hamburg-Eppendorf, Hamburg, Germany; 10 Stroke Trials Unit, Division of Clinical Neuroscience, University of Nottingham, Nottingham, UK

**Keywords:** Stroke, regulatory approval, randomised clinical trial

## Abstract

**Introduction:**

The initiation and conduct of randomised clinical trials are complicated by multiple barriers, including delays in obtaining regulatory approvals. Quantitative data on the extent of the delays due to national or local review in randomised clinical trials is scarce.

**Materials and methods:**

We assessed the times needed to obtain regulatory approval and to initiate a trial site for an academic, EU-funded, phase III, randomised clinical trial of pharmacological prevention of complications in patients with acute stroke in over 80 sites in nine European countries. The primary outcome was the time from the first submission to a regulatory authority to initiation of a trial site. Secondary outcomes included time needed to complete each individual preparatory requirement and the number of patients recruited by each site in the first 6 and 12 months.

**Results:**

The median time from the first submission to a regulatory authority to initiation of a trial site was 784 days (IQR: 586–1102). The single most time-consuming step was the conclusion of a clinical trial agreement between the national coordinator and the trial site, which took a median of 194 days (IQR: 93–293). A longer time to site initiation was associated with a lower patient recruitment rate in the first six months after initiation (B = –0.002; *p* = 0.02).

**Discussion:**

**Conclusion:**

In this EU-funded clinical trial, approximately 26 months were needed to initiate a trial site for patient recruitment. The conclusion of a contract with a trial site was the most time-consuming activity. To simplify and speed up the process, we suggest that the level of detail of contracts for academic trials should be proportional to the risks and commercial interests of these trials.

## Introduction

Randomised clinical trials (RCTs) and meta-analyses thereof are generally considered the best instruments to assess whether a specific diagnostic test or treatment is of benefit to patients or healthy persons,^1^ but their initiation and conduct are hampered by multiple barriers. Editorials and narrative reviews have reported lack of funding, increasing complexity of regulations, excessive monitoring, overinterpretation of privacy laws, and complex and overly bureaucratic trial procedures, often out of proportion to the conceivable risk to research participants, as important obstacles.^2–5^ In addition, delays in obtaining ethical, regulatory, and legal approvals have been identified as major delaying factors in initiating clinical trials sites.^2^ As a result of these and other barriers, it has been estimated that approximately half of the clinical trials fail to reach their target sample size within the planned timeline.^6^

Quantitative evidence on the true extent of the delays in RCTs due to institutional or legal review is scarce and limited to a specific part of the approval process, to specific countries and time periods.^5,7,8^ New regulations, such as the General Data Protection Regulation in the European Union, have been introduced in recent years, which could have major consequences for institutional review and contractual governance.

In the present study, we aimed to quantify delays caused by legal or institutional review in the PRECIOUS trial (PREvention of Complications to Improve OUtcome in elderly patients with acute Stroke). This is a multicentre, multinational clinical trial, performed in over 80 sites in 9 European countries, and supported by the European Union’s Horizon 2020 programme.^9^ We provide a systematic overview of the time period required for each regulatory approval procedure needed to open an individual trial site and analyse its relationship with patient recruitment.

## Methods

PRECIOUS is an international, multi-centre, 3*2-factorial, randomised, controlled, open-label clinical trial with blinded outcome assessment (PROBE) of the preventive use of metoclopramide, ceftriaxone, paracetamol, or any combination of these, for four days in elderly patients with acute ischaemic stroke or intracerebral haemorrhage. The trial was initiated in 2015 and aims to recruit 3800 patients in about 80 hospitals (both academic and general) in 9 European countries: Estonia, Germany, Greece, Hungary, Italy, the Netherlands, Norway, Poland, and the UK. An overview of the regulatory requirements for starting the trial on international, national, regional and local levels is provided in Table 1 and described in more detail below.

**Table 1.** Overview of regulatory requirements for each country.

Country	International	National			Regional/local			
	VHP	EC^a^	NCA	CCA	CTA	HEC/REC	Hospital^b^	SIV
Estonia	×	×	×	×	×	–	×	×
Germany	×	×	×	×	×	×^c^	–	×
Greece	×	×	×	×	×	×	×	×
Hungary	×	×	×	×	×	×^c^	–	×
Italy	×	×	×	×	×	×	×	×
Norway	–	×	×	×	×	–	–	×
The Netherlands	–	×	×	×	×	–	×	×
Poland	–	×	×	×	×	×^c^	–	×
United Kingdom	×	×	×	×	×	×^d^	×	×

VHP: voluntary harmonisation procedure; CCA: Country Coordinator Agreement; EC: national ethics committee; NCA: National Competent Authority; CTA: clinical trial agreement; REC: regional ethics committee; HEC: hospital ethics committee; SIV: site initiation visit.

^a^The EC is the national ethics committee of a country or the leading ethics committee affiliated the hospital of the national coordinator.

^b^Hospital stands for local hospital approval.

^c^No separate submission is required, the EC or NCA contacts the REC for approval during the approval process.

^d^Review by the UK’s NHS Research Scotland (NRS) and Health Research Authority (HRA) were categorised under REC.

### Overview of preparatory requirements

On an international level, we requested a ‘Voluntary Harmonisation Procedure’ (VHP) for six countries (Estonia, Germany, Greece, Hungary, Italy and the United Kingdom). The VHP provides a coordinated assessment of a clinical trial application in multiple European countries.^10^ Three countries were not included in the VHP (Netherlands, Norway and Poland).

After VHP approval, subsequent evaluation on a national level was needed by the National Competent Authority (NCA) and leading or national Ethics Committee (EC) in each country. The leading EC was usually the EC affiliated to the hospital of the National Coordinator (NC), who is the coordinating investigator for each country. In some countries (Greece, Italy), an independent review by an Ethics Committee on a regional (REC) or hospital (HEC) level was also required, as well as approval from each participating site (usually given by the Board of Directors) was needed to endorse the practicability of conducting the trial at that site in some countries (Table 1).

In addition and often in parallel, two types of required legal documents were completed: a Country Coordinator Agreement (CCA) and a Clinical Trial Agreement (CTA). A CCA is a contract signed between the trial sponsor, University Medical Center Utrecht (UMCU) in the Netherlands, and the institution of the NC of each participating country, which delegated the responsibility for arranging legal agreements for that country from the sponsor to the NC, in order to prevent potential problems due to different national laws between the participating and the Sponsor’s country. Subsequently, the institution of the NC contracted each participating trial site in that country by means of a CTA.

After obtaining all necessary regulatory approvals and completing all contracts, the Site Initiation Visit (SIV) was planned. During this meeting with the local PRECIOUS team, a national trial monitor assessed whether all mandatory preparations had been completed and whether the Investigator Site File contained a copy of all the necessary documents (e.g. approval letters of the regulatory authorities, lists of signatures and CVs of trained site PRECIOUS personnel). After approval of the SIV report by the central monitoring team of European Clinical Research Infrastructure Network, a site was considered ready to start recruiting patients.

### Included sites and data collection

We included a trial site in this analysis if the start and end dates (‘milestone dates’) for one or more of the individual regulatory preparatory processes were available: VHP, EC, NCA, REC/HEC, local hospital approval, CCA or CTA. A trial site was excluded if there were specific circumstances that resulted in exceptional delay in opening the site (e.g. long-term sick leave of the principal investigator that delayed all regulatory approvals). Two authors (JCdJ, HR) retrospectively retrieved milestone dates from correspondence with regulatory authorities (e.g. approval letters) and signed contracts stored in the Trial Master File during the trial. The start of contract negotiations was retrieved from email correspondence and supplemented by information from the relevant national research teams. For each site we collected the number of included patients in the first 6 and 12 months after the date of the SIV from the study’s electronic case file.

### Measures and outcomes

We distinguished trial sites that were included in the original submissions to the national authorities (‘original sites’) and trial sites that expressed interest in joining during the course of the trial, which had to be added by means of an amendment because national approvals were already obtained (‘additional sites’).

The primary outcome was the time needed to initiate the original trial sites (‘time to site initiation’), which was defined as the time period between the date of VHP submission and SIV approval (for VHP countries), between submission to the EC and SIV approval (for the Netherlands) or between sending the CCA template to the NC and SIV approval (for Norway and Poland, where this was the first preparatory activity).

Secondary outcomes included (1) time to site initiation for additional sites; (2) time needed to complete each individual preparatory requirement; (3) average time to site initiation in each of the 9 participating countries; (4) average time to site initiation in academic and non-academic hospitals; and (5) number of patients recruited by each site in the first 6 and 12 months after SIV. Time to site initiation for additional sites was defined as the time between the date of the first preparatory activity (either applying for an amendment to the EC or sending the CTA template to the hospital) and the SIV date (defined as the last signature on the SIV report), which means that the time needed to obtain primary (inter)national approvals (VHP, EC, NCA, CCA) was not included in this outcome. The time needed for individual regulatory approvals (VHP, EC, NCA, REC/HEC, local hospital approval) was defined as the time period between submission date to the regulatory authority and their approval. The time needed for concluding a contract (CCA, CTA) was defined as the time period between the date the first draft version of the contract was sent to the trial site (i.e. national coordinator or institution lawyer), and the date of the last signature on the contract. As some regulatory processes may be done in parallel or may overlap, the time to site initiation is not necessarily the sum of the time periods needed for all individual preparatory requirements.

### Statistical analysis

The time to site initiation and the time period needed for each regulatory requirement to be completed are reported in median days with interquartile range. Differences in time to site initiation between original and additional sites, and between academic and non-academic hospitals were analysed with the Mann–Whitney U test. Differences between countries were assessed with one-way non-parametric ANOVA (Kruskal–Wallis test). The association between median time to initiation and patient recruitment in the first 6 and 12 months was assessed with linear regression. The criterion for statistical significance was set at α = 0.05. The data that support the findings of this study are available from the corresponding author upon reasonable request.

## Results

**Figure 1.**
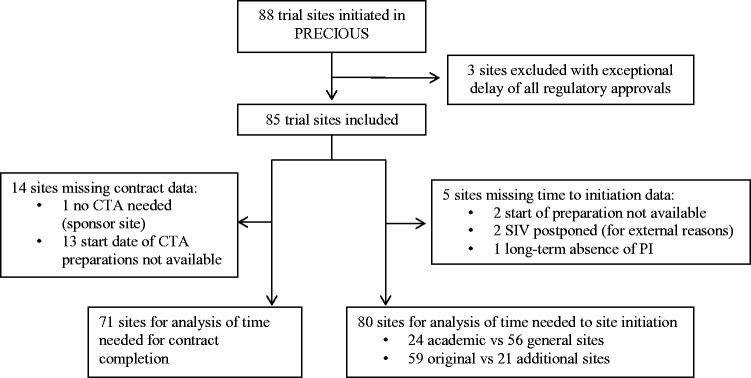
Flowchart of included trial sites in the current study.

During the course of PRECIOUS, 88 trial sites planned to participate in the trial. In the present study, three trial sites were excluded because all of the regulatory approvals of these sites were delayed because of extraordinary circumstances. Therefore, 85 trial sites were included in the analysis (Figure 1). The start date of PRECIOUS was submission to the Sponsor’s EC on 16 October 2015.

The median time to initiation was available for 80 sites; 59 of 60 (98%) original sites (one UK site excluded because of delay of the SIV due to long absence of the PI) and 21 of 25 (84%) additional sites. Two additional sites were excluded (both in Italy), because the start date of the preparation period was not available, and two sites (one in Germany, one in UK) were excluded because the SIV was postponed for other reasons (Figure 1). The median time to site initiation was 784 days (IQR: 586–1102) for the 59 original trial sites and 234 days (IQR: 166–315; *p* < 0.0001) for the 21 additional trial sites. For original and additional trial sites combined, the median time to initiation was 698 days (IQR: 409–979; Table 2).

**Table 2.** Time to site initiation per country.

	Original sites			Additional sites		
Country	Number of sites	Median (IQR)	Range	Number of sites	Median (IQR)	Range
Netherlands	10/10	504 (437–551)	238–700	4/4	175 (166–325)	166–372
Estonia	4/4	591 (573–670)	569–695	–	–	–
Norway	4/4	735 (574–971)	545–1025	–	–	–
United Kingdom	6/7	760 (718–903)	716–987	12/13	212 (163–256)	91–566
Germany	6/6	767 (605–925)	567–1096	1/2	406	–
Greece	3/3	793 (774–821)	774–821	2/2	308	287–329
Italy	9/9	813 (702–1115)	543–1131	0/2	–	–
Poland	6/6	956 (812–1108)	711–1113	1/1	116	–
Hungary	13/13	1235 (1201–1285)	564–1430	1/1	1324	–
Total	59/60	784 (586–1102)	238–1430	21/25	234 (166–315)	91–1324

Notes: Time is displayed as days. Countries sorted on duration of time to initiation.

IQR: interquartile range.

**Figure 2.**
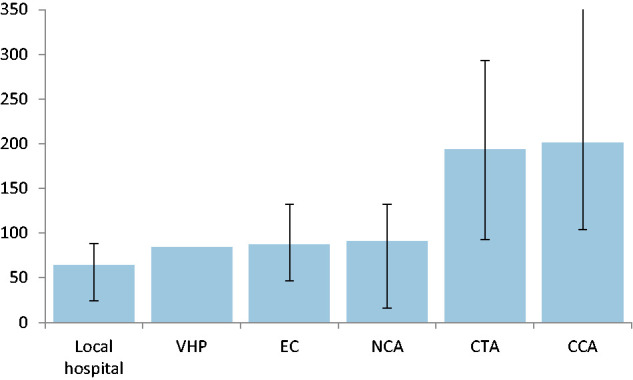
Median time per regulatory requirement. Approval duration of national regulatory requirements sorted based on duration. Time displayed in median number of days with interquartile ranges.

The time needed for each separate regulatory requirement to be completed is shown in Tables 3 and 4. The median EC and NCA review time was 87 (IQR: 37–128) and 91 (IQR: 16–132) days, respectively. For VHP countries, the review by the NCA lasted 105 (IQR: 10–156) days. The signing process of the CCA and CTA were the most time-consuming regulatory requirements (Figure 2**)**. The median time needed to sign a CCA was 201 days (IQR: 104–492) and to sign a CTA was 194 days (IQR: 93–293). On average, 35.9% of the time to initiation was used for signing the CTA.

**Table 3.** Time needed for each (inter)national regulatory approval.

Country	VHP	EC	NCA	CCA
Estonia	84	44	111	376
Germany	84	149	14	74
Greece	84	57	99	52
Hungary	84	87	153	768
Italy	84	28	163	193
Netherlands	–	110	21	–
Norway	–	136	91	192
Poland	–	29	88	531
United Kingdom	84	119	8	209
Median (IQR)	–	87 (37–128)	91 (16–132)	201 (104–492)

Notes: Time is displayed in days. The approval time is the time of the first approval (amendments for adding sites are not included).

EC: ethics committee; NCA: National Competent Authority; CCA: Country Coordinator Agreement; IQR: interquartile range; VHP: voluntary harmonisation procedure.

**Table 4.** Time needed for each local regulatory approval.

	CTA		Local hospital approval		REC	
Country	Number of sites	Median (IQR)	Number of sites	Median (IQR)	Number of sites	Median (IQR)
Estonia	4/4	105 (52–130)	3/4	13 (5–66)	–	–
Germany	8/8	542 (231–779)	–	–	–	–
Greece	5/5	208 (198–281)	4/5	69 (65–82)	5/5	41 (23–61)
Hungary	13/13	328 (277–368)	–	–	–	–
Italy	1/11	51	4/11	39 (6–152)	10/11	51 (24–67)
Netherlands	13/14^a^	140 (60–179)	13/14^a^	25 (10–57)	–	–
Norway	1/4	81	–	–	–	–
Poland	6/6	205 (157–252)	–	–	–	–
United Kingdom	20/20	118 (70–196)	20/20	85 (39–108)	20/20	39 (4–131)
Total	71/85	194 (93–293)	44/54	61 (22–88)	35/36	41 (14–69)

Note: Time is displayed in days.

IQR: interquartile range; CTA: Clinical Trial Agreement; REC: regional ethics committee.

^a^Since the UMCU was the coordinator centre, no CTA or local hospital approval had to be obtained.

The median time to initiation was similar for academic (*n* = 24) and general (*n* = 56) hospitals (659 vs 703 days, *p* = 0.77). The median time to initiation differed significantly between countries (*p <* 0.0001), with the shortest time to initiation for the Netherlands (where approval was requested first) and the longest for Hungary (see Table 2). A longer time to initiation was associated with a slower patient recruitment in the first six months after initiation (B = –0.002; *p* = 0.02), but not in the first 12 months (B = –0.003; *p* = 0.12; see Supplementary Tables 1 and 2).

## Discussion

In PRECIOUS, the median time to initiation of a pre-planned trial site was just over two years. Negotiations on contracts between a national coordinator and trial sites were an important delaying factor, responsible for approximately one third of the time needed to initiate a trial site.

PRECIOUS is an investigator-initiated, pragmatic clinical trial testing widely available off-patent medications that have been on the market for several decades and that have proven to be safe in stroke patients.^11,12^ As a consequence, the trial was considered as low risk by regulatory authorities. The study Sponsor had almost full-time availability of a chief investigator, trial coordinator, and research nurse to support the submissions and applications. The trial was coordinated in the participating countries by experienced NCs and research teams. In three countries (Greece, Hungary, and Poland), a clinical research organisation (CRO) was contracted during the course of the trial to speed up the approval process. Nevertheless, we experienced considerable delays in obtaining ethics and hospital management approvals. Considering that the duration of the trial granted by the European Union was 60 months, more than half of the time intended to recruit patients was spent on obtaining regulatory approvals. In addition, we found that longer time to site initiation was negatively associated with the number of included patients in the first six months, possibly because of understandable loss of momentum and enthusiasm among some of the investigators. Delays in obtaining regulatory approval and legal review may therefore be an important reason why about half of the clinical trials fail to reach their target sample size within the planned timeline.^6^

Only a few previous studies evaluated delay due to regulatory review in RCTs.^5^ Also, most of these studies only looked at ethics^13,14^ or local hospital approval,^8^ instead of the entire time to initiation including contract negotiations. In the British phase IV trial SANADII on treatment of epilepsy the median ‘opening time’ for study sites in 2012 and 2013 was 10.5 months, but this was after ethics approval had already been obtained.^7^ The study identified several delaying factors, such as negotiating excess treatment costs, finalising logistics, collecting CVs, and ongoing discussions about participation. The median time of 10.5 months is much shorter than the median time to initiation of almost 25 months for pre-planned trial sites in the UK, but the starting point of VHP submission in the present study is much earlier. In addition, SANADII was a phase IV trial whereas PRECIOUS is phase III, and SANADII was performed only in the UK, whereas PRECIOUS involves nine European countries.

Obviously, we have to declare a mea culpa. Although the study Sponsor and most of the centres of the NCs have ample experience with clinical trials, and all of these are partners in the PRECIOUS project and therefore share responsibilities, they may occasionally have contributed to some delays, for example because of other obligations or priorities. This also applies to local Principal Investigators. Most investigators work on the trial in addition to their everyday clinical work. The trial had no commercial interest and local investigators only receive a small reimbursement for expenses for including a patient in the trial, which could have an impact on the speed of setting up the trial. With the exception of Greece, Hungary, and Poland, the approval process was not supported by a commercial CRO, which could have accelerated the process but at considerable cost. It would be interesting to compare our findings with those in other academic trial with or without the involvement of a CRO, and with those in industry-driven trials. Such a comparison could support the development of best practice examples that could aid future clinical trials.

The current study also has other limitations. First, the time needed for preparation of the submissions to the regulatory authorities is not incorporated in the duration of the approval processes. The submission package consists of multiple forms and other documents, some of which need translation to the national language in some countries. Therefore, the actual time needed for ethics or hospital management approval is longer than reported here. Secondly, we assessed the approval process in just nine countries in Europe. These were however relatively well distributed across Europe.

We observed considerable overlap between the assessments of different regulatory authorities. Whereas the VHP is intended to facilitate and shorten NCA approval and VHP timelines dictate that NCA approval should follow within 10 days after VHP approval,^10^ we experienced a median time for NCA review of 105 days in the six countries that participated in the VHP (in addition to the 84 days needed for VHP approval). Therefore, in our experience the VHP was an additional bureaucratic burden that required a separate submission with often little additional value with regard to subsequent approvals. We believe that stricter adherence to VHP timelines should be pursued. Likewise, the EC provides ethical approval for a clinical trial and ensures that a trial is performed according to (inter)national law and regulations. This approval should serve as a proof of warranty for local trial sites to conduct a clinical trial. However, in our experience, institutions at regional or hospital level may repeat a large part of the same approval procedure as the EC. In our opinion, countries should strive for a single regulatory review process that serves as global approval in that country. Any local review afterwards should be limited to issues related to local practicability and should be bound to specific timeframes. In addition, we support proposals to make regulatory requirements proportional to the risk of the study. This is likely to shorten the approval process and to increase patient recruitment.

Moreover, clinical trials could benefit from a universally accepted template for national contracts. In PRECIOUS, we used a local template for the CCAs. The CCA was reviewed by lawyers in each country, who were often not familiar with this format. In addition, there are no established timeframes for legal review of research contracts (both CCA and CTA). Legal departments of hospitals or institutions often have a high work load with limited capacity and the quick opening of a trial site may not be their top priority. This regularly resulted in recurrent discussions between lawyers of both parties, often on details of which the relevance was not immediately clear to the investigators, interrupted by lengthy periods of apparent inactivity. This is illustrated by a delay of 201 days for the CCA and 194 days for the CTA. Moreover, most of the times the CCA and CTA were handled consecutively. We suggest that an international template for clinical trials should be developed, with specific timeframes and deadlines for local lawyers to complete legal review.

In conclusion, ethical and legal review including the evaluation of contracts with study sites lead to serious delays in initiating trial sites, which reduce time available for patient recruitment, and results in substantial increases in efforts and costs, jeopardising the conduct of academic clinical trials.

## Supplemental Material

sj-pdf-1-eso-10.1177_23969873211004845 - Supplemental material for Regulatory delays in a multinational clinical stroke trial Click here for additional data file. Supplemental material, sj-pdf-1-eso-10.1177_23969873211004845 for Regulatory delays in a multinational clinical stroke trial by Jeroen C de Jonge, Hendrik Reinink, Bridget Colam, Iris Alpers, Alfonso Ciccone, Laszlo Csiba, Janika Kõrv, Iwona Kurkowska-Jastrzebska, Malcolm R Macleod, George Ntaios, Götz Thomalla, Philip M Bath and H Bart van der Worp in European Stroke Journal
