# Supplementary material for: Regulatory delays in a multinational clinical stroke trial
Source: Eur Stroke J. 2021 Mar 30;6(2):120–7. doi: 10.1177/23969873211004845 (PMC8370076; doi:10.1177/23969873211004845)
Supplement: sj-pdf-1-eso-10.1177_23969873211004845 - Supplemental material for Regulatory delays in a multinational clinical stroke trial [file sj-pdf-1-eso-10.1177_23969873211004845.pdf]

Supplementary Table 1 – Time to first included patient

Country	REC	
	**Number of sites**	**Median [IQR]**
Estonia	4/4	573 [251-692]
Germany	6/8	63 [14-148]
Greece	5/5	66 [29-132]
Hungary	7/13	72 [7-204]
Italy	9/11	89 [30-216]
Netherlands	11/14	71 [26-162]
Norway	2/4	271
Poland	5/6	88 [19-172]
United Kingdom	18/20	47 [24-108]
Total	67/85	83 [33-154]

Median number of days from trial site initiation to the first included patient for each country
Abbreviations: IQR, interquartile range.
